# Slaughterhouses sustainability analysis in special capital region of Jakarta Province, Indonesia

**DOI:** 10.14202/vetworld.2019.748-757

**Published:** 2019-06-04

**Authors:** H. A. Sidabalok, Macfud Macfud, N. Ramli, N. K. Pandjaitan

**Affiliations:** 1 Study Program of School of Environment Science, IPB Graduate School, Bogor Agricultural University, Bogor, Indonesia; 2 Department of Agroindustrial Technology Bogor Agricultural University, Bogor, Indonesia; 3 Department of Nutrition and Feed Science Bogor Agricultural University, Bogor, Indonesia; 4 Department of Communication and Community Development Bogor Agricultural University, Bogor, Indonesia

**Keywords:** attitude, knowledge, practice, slaughterhouse, sustainability

## Abstract

**Aim:**

The objective of this research was understanding slaughterhouses sustainability and the prospection status in special region of Jakarta Province Indonesia.

**Materials and Methods:**

The concept of sustainable slaughterhouse was formed based on social, economy, ecology, technology, and institutional dimension. Research objects were three types of slaughterhouses in Special Capital Region of Jakarta Indonesia; pig slaughterhouse, chicken slaughterhouse, and ruminant slaughterhouse. Tools used were questionnaires to assess the perception of people living around slaughterhouses, assessment of the knowledge, attitude, and practice from slaughterhouse management, along with assessment and focus group discussion for sustainability test. Methods used were descriptive analysis and sustainability test by multidimensional scaling method. Data collected consisted of primary and secondary data. Primary data were obtained by field survey, interview, questionnaire, measurement of the waste threshold, and microbe contamination, whereas secondary data were obtained from slaughterhouse agency. Data were analyzed with IBM statistical package for the social sciences (SPSS^Ò^) version 18 to calculate characteristic, variables correlation, sustainability test with Rapfish^Ò^ modified into Rap slaughterhouse, and prospective analysis with PPA.

**Results:**

The level of sustainability for pig slaughterhouse was moderately sustainable with 0.5173 index value, ruminant slaughterhouse was moderately sustainable with 0.5171 index value, and chicken slaughterhouse was moderately unsustainable with 0.4530 index value.

**Conclusion:**

Scenario on policies that should be applied in ruminant slaughterhouse was increasing the use of waste as biogas; for chicken slaughterhouse was increasing promotion and for pig slaughterhouse was increasing product quality control. The implication of this research was to provide input based on a scientific study for the local government of Jakarta in managing the slaughterhouses.

## Introduction

Based on The Center of Statistical Data (Badan Pusat Statistik) [1] for Jakarta Province, the population of Jakarta in 2017 was 10,377,924 peoples. In 2017, the demand for beef, chicken meat, and pork in Jakarta were 60.376 tonnes, 365.000 tonnes, and 1.000 tonnes, respectively. Only 20% of the demand could be fulfilled by slaughterhouses in Jakarta, which were ruminant slaughterhouse in Cakung, pig slaughterhouse in Kapuk, and chicken slaughterhouse in Rawa Kepiting. The rest of the demand was fulfilled by slaughterhouses outside of Jakarta.

Based on Law Number 18 of 2009 concerning Animal Husbandry and Animal Health and its amendments to Law No. 41 of 2014 Article 6, which requires slaughter of animals whose meat is circulated to be carried out in slaughterhouses and must follow slaughter methods that meet veterinary public health rules and animal welfare. The implementation of the Law is elaborated through Government Regulation of the Republic of Indonesia Number 95 of 2012 concerning Public Health Veterinary and Animal Welfare, which regulates the operation of slaughterhouses.[2]. Animal slaughter industry in principle is a way to improve living standard and economy although the waste may cause an inevitable negative impact on environmental balance. Good hygiene and sanitation are implemented to suppress bacterial growth in an indicator of the existence of fecal and pathogenic bacterial contamination, which needed improvement [3]. Environmental management is done to prevent a negative impact on public health be it to people who consume the product originating from animal slaughterhouses or to people who live near animal slaughterhouses [4].

Researches on sustainability slaughterhouse needed to be done so that slaughterhouses management can implement sanitary hygiene practices and good waste management, orienting on profit by considering social and public health aspect, technology implementation and dynamic institutional coordinating [5].

The objective of this research was to analyze the sustainability of slaughterhouses especially in three slaughterhouses: Ruminant slaughterhouse, poultry slaughterhouse, and pig slaughterhouse based on social, economic, ecological, technological, and institutional dimension, and then determining policy scenario for sustainable slaughterhouse implementation in Jakarta Province Indonesia.

## Materials and Methods

### Ethical approval

The approval to conduct this study was obtained via the following permissions: permission from DKI Jakarta Provincial Health Office with the approval number, 672/SDK/VI/2017; a letter of recommendation from the chairman of the study program of natural resource management and environment of Bogor Agricultural University, with the SK number 316/IT3.10.2/KM/2017.

### Informed consent of participants

Informed consent was obtained from all the participants/respondents.

### Research time and location

The research was done between April 2018 and August 2018 in the following locations: (1) Cakung for ruminant slaughterhouse; (2) Rawa Kepiting for poultry slaughterhouse; and (3) Kapuk for pig slaughterhouse. Slaughterhouse determination was done by purposive sampling based on certain criteria.

### Research concept framework

Slaughterhouse sustainability analysis in Jakarta Province was compiled out of several dimensions: Social (S), economical (Ec), environmental (En), institutional (I), and technological (T) dimension. These attributes consisted of characteristics and perceptions from people around slaughterhouse, the management of slaughterhouse, stakeholders of slaughterhouse, academician/professionals, and observation results in the form of primary and secondary data. The assessment result from these attributes would be the sustainability dimension assessment value measured by multidimensional scaling (MDS) method. Afterward, the prospective analysis was done to observe the main triggering attribute in managing sustainable slaughterhouse using participatory prospective analysis (PPA) method. This status was used as the basis of policy scenario so that sustainable management of animal slaughterhouse could be achieved.

### Respondent and data source

There were 108 local resident respondents from all slaughterhouses, which consisted of 40 respondents from ruminant slaughterhouse, 43 respondents from chicken slaughterhouse, and 25 respondents from pig slaughterhouse. The respondent’s criteria were influential residents or head of family within 1 km radius of the slaughterhouse [6]. The number of slaughterhouse worker respondents was 108 respondents with 45 respondents from ruminant slaughterhouse, 33 respondents from chicken slaughterhouse, and 30 respondents from pig slaughterhouse with sample size depending on the proportion of the number of workers in every slaughterhouse, which may comprise butchers, waste worker, health worker, and management worker. Sample gathering technique to acquire information and knowledge from stakeholders and professionals used expert survey method through in-depth interview and focus group discussion under purposive sampling to determine dimension, data sources and references for sustainable slaughterhouse management (Table-1). Respondents consisted of PD. Dharma Jaya Chief Director, head of Puskesmas (local government clinic), the head of the animal husbandry, the head of slaughterhouse management section, and professional/academician. Questionnaire respondents for PPA were PD Dharma Jaya Director, the head of food security, marine, and agriculture agency of Jakarta Province, the head of Jakarta Province economy bureau, the head of animal husbandry division, the head of slaughterhouse, and competent professional/academician.

**Table-1:** Dimension, data source and references for sustainable slaughterhouse management.

Code	Environmental dimension attributes	Data source	References
En1	Recording of number of slaughters every day	Observation	[7-10]
En2	Temporary shelter and slaughter area cleaning frequency	Observation	
En3	Waste pipe/drainage leakage	Observation	
En4	Waste and rain water separation	Observation	
En5	Waste control with debt measuring device	Observation	
En6	Separation of liquid and solid waste	Observation	
En7	Incinerator	Observation	
En8	Waste usage as organic fertilizer or biogas	Observation	
En9	Waste threshold control	Laboratory test and data from management	
En10	General knowledge of slaughterhouse waste management	Questionnaire	
En11	Soil pollution	Questionnaire	
En12	Flood location	Questionnaire	

**Code**	**Economy dimension attributes**	**Data source**	**References**

Ec1	Region retribution target	Secondary data	[1,11,12]
Ec2	Market share	Secondary data	
Ec3	Slaughterhouse product marketing coverage	Secondary data	
Ec4	Demand level	Secondary data	
Ec5	Promotion	Secondary data	
Ec6	Product health control	Questionnaire	
Ec7	Livestock supply sustainability	Secondary data	
Ec8	Livestock life quality control	Questionnaire	
Ec9	Businesses other than meat industry	Secondary data	
Ec10	People’s economical perception	Questionnaire	
Ec11	Slaughterhouse worker/management learning level	Questionnaire	

**Code**	**Social dimension attributes**	**Data source**	**References**

S1	Health status of slaughterhouse worker	Questionnaire	[6,7,13,14]
S2	Disease prevalence in people	Secondary data	
S3	Slaughterhouse microbiological sample examination	Lab examination and secondary data	
S4	Worker level of education	Questionnaire	
S5	Slaughterhouse acceptance perception of local people	Questionnaire	
S6	Waste-related disease in local people	Questionnaire	
S7	Disease frequency	Questionnaire	
S8	Slaughterhouse management level of knowledge on hygiene, sanitation, and waste management	Questionnaire	
S9	Slaughterhouse management concerning hygiene, sanitation, and waste management	Questionnaire	
S10	Slaughterhouse management practice on hygiene, sanitation, and waste management	Questionnaire	
S11	Slaughterhouse worker training frequency on slaughterhouse worker as profession	Questionnaire	

**Code**	**Technology dimension attributes**	**Data source**	**References**

T1	Biogas technology implementation	Observation	[7,15,16]
T2	Organic fertilizer technology implementation	Observation	
T3	GHP implementation	Questionnaire	
T4	Cold chain implementation in product distribution	Secondary data	
T5	Slaughter system characteristic	Observation	
T6	Slaughterhouse product diversification	Secondary data	

**Code**	**Institutional dimension attributes**	**Data source**	**References**

I1	Slaughterhouse location suitability with general layout plan of Jakarta	Secondary data	[17,18]
I2	Slaughterhouse operational alliance	Secondary data	
I3	Coordination pattern with livestock supplier region	Interview	
I4	Cooperation with businesses from other regions	Interview	
I5	Transactional coordination pattern	Interview	
I6	Productive female livestock slaughter ban policy (ruminants)	Questionnaire	
I7	Live livestock import policy (ruminant)	Interview	

GHP=Good hygiene practices

### Statistical analysis

Data analysis consisted of several stages, which were situational analysis from primary and secondary data followed by multidimensional analysis (MDS) with Rapfish software, which was modified into Rap slaughterhouse. Rapfish is a statistical technique for rapid appraisal of relative status of entities (=fisheries), judged quantitatively against pre-defined sets of attributes grouped into “evaluation fields” or disciplines. The Rapfish technique is flexible such that other modalities of status may be used, such as conformity with a set of specified objectives or compliance with a code of conduct. Multidimensional scaling analysis consisted of ecology, economy, social, technology, and institutional of every slaughterhouse. Attributes from dimension were scored based on the real data condition in the field be it from interview and observation (primary data) or using secondary data. Scoring was based on references from literature and judgment in accordance with scientific assumptions and principles. Scores obtained were then inputted into excel with previously prepared template and then processed until Rap slaughterhouse value was obtained, called sustainability index. The sustainability index value was separated into four levels, 0-25 range was within bad/unsustainable status, 26-50 range was fairly unsustainable, 51-75 range was moderately sustainable, and 76-100 was within sustainable [19].

The most influential attributes in every dimension were arranged in a questionnaire to determine the effect and dependence of every attribute through a prospective test to obtain essential factor for policy scenario in developing the current studied system. The method used was PPA through pairwise comparison approach to see the effect and dependence of every attribute between each other to obtain measured global value from every dimension considered as main attributes [20].

## Results

### Multidimensional scaling and sustainability validation analysis in ruminant slaughterhouse

**Figure-1:**
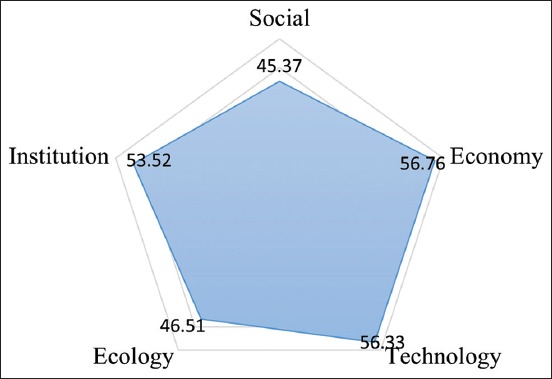
Kite diagram of ruminant slaughterhouse.

Analysis result of MDS method in ruminant slaughterhouse generated the sustainability value of every dimension. Economy, institutional, and technology dimension sustainability status were considered as moderately sustainable with sustainability value >50 with economy dimension being 56.76, institutional dimension being 53.52, and technology dimension being 56.33. Dimensions on fairly sustainable category were ecology dimension with 46.51 and social dimension with 45.37; thus, efforts are needed to improve sustainability in ecology and social dimension. Visualization of kite diagram to illustrate the sustainability status between dimensions is observed in Figure-1.

The validity of MDS analysis was confirmed by Monte Carlo analysis. The gap between MDS analysis result with Monte Carlo comparison value was <5% or really small, which means that the level of faults that may influence MDS process was >5%, that the influence of faults to attributes scoring was very small (Table-2).

**Table-2:** Calculation of sustainability index and validation in ruminant slaughterhouse.

Dimension	Dimension status	Monte Carlo	Delta	RSQ	Stress
Social	45.37	46.06	0.69	0.9486	0.1320
Economy	56.76	56.05	0.71	0.9533	0.1348
Ecology	46.51	46.23	0.28	0.9534	0.1368
Institution	53.52	53.66	0.14	0.9393	0.1482
Technology	56.33	56.20	0.13	0.9433	0.1490

RSQ=Responses to stress questionnaire

### MDS and sustainability validation analysis in chicken slaughterhouse

**Figure-2:**
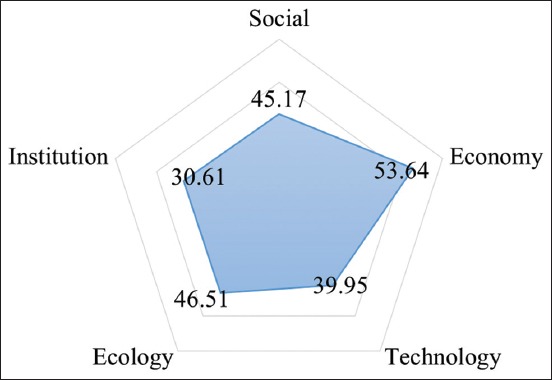
Kite diagram of chicken slaughterhouse.

Analysis result using MDS method in chicken slaughterhouse showed the sustainability value of every dimension. Sustainability status of economy dimension was categorized as moderately sustainable with 53.64; however, the rest of the value were included as fairly sustainable with social dimension being 32.50, ecology dimension being 46.51, institutional dimension being 30.61, and technology dimension being 39.95 (Figure-2).

Validity of MDS analysis was confirmed by Monte Carlo analysis. The gap between MDS analysis result with Monte Carlo comparison value was <5% or really small, which means that the level of faults that may influence MDS process was >5% (Table-3). From the analysis, it can be summarized that the influence of faults to attributes scoring was very small.

**Table-3:** Calculation of sustainability index and validation in chicken slaughterhouse.

Dimension	Dimension status	Monte Carlo	Delta	RSQ	Stress
Social	32.50	34.56	2.06	0.9471	0.1316
Economy	53.64	53.34	0.30	0.9517	0.1303
Ecology	46.51	46.23	0.28	0.9534	0.1368
Institution	30.61	32.32	2.71	0.9478	0.1376
Technology	39.95	41.64	1.69	0.9334	0.1434

RSQ=Responses to stress questionnaire

### MDS and sustainability validation analysis in pig slaughterhouse

**Figure-3:**
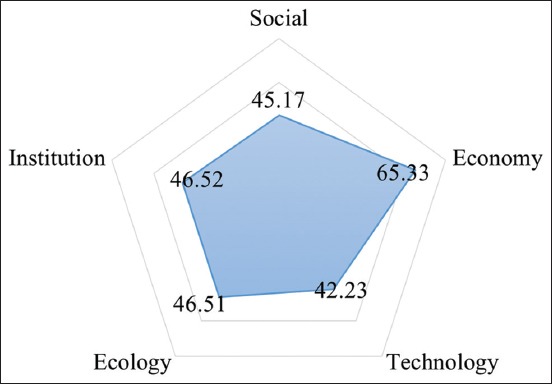
Kite diagram of pig slaughterhouse.

Analysis result using MDS method in pig slaughterhouse showed the sustainability value of every dimension. The sustainability status of economy dimension was categorized as moderately sustainable with 65.33, while other dimensions were categorized as not so sustainable with social dimension being 45.17, ecology dimension being 46.51, institutional dimension being 46.52, and technology dimension being 42.23 (Figure-3).

The validity of MDS analysis was confirmed by Monte Carlo analysis. The gap between MDS analysis result with Monte Carlo comparison value was <5% or really small, which means that the level of faults that may influence MDS process is >5%. From the analysis, it can be summarized that the influence of faults to attributes scoring was very small (Table-4).

**Table-4:** Complete calculation of sustainability index and validation in pig slaughterhouse.

Dimension	Dimension status	Monte Carlo	Delta	RSQ	Stress
Social	45.17	45.62	2.06	0.9490	0.1317
Economy	65.33	64.03	0.30	0.9535	0.1309
Ecology	46.51	46.23	0.28	0.9534	0.1368
Institution	46.52	46.49	2.71	0.9383	0.1550
Technology	42.23	42.47	1.69	0.9299	0.1473

RSQ=Responses to stress questionnaire

### Animal slaughterhouse sustainability analysis

**Figure-4:**
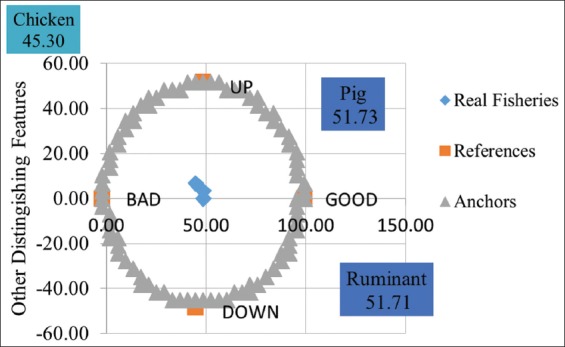
Sustainability status of every slaughterhouse.

In general, economy dimension was grouped as moderately sustainable (>50). This indicated that every slaughterhouse must be benefitting economically, while dimension with the least sustainability was social dimension (between 25 and 50) since many people did not agree with the existence of slaughterhouse and many also suffered diseases caused by slaughterhouse waste. In general, ruminant and pig slaughterhouse were grouped as sustainable with sustainability index ranging between 51 and 75 and chicken slaughterhouse was grouped as less sustainable with index ranging 26-50 (Figure-4).

Sustainability in every dimension was affected by a leverage factor. Leverage factor is the most sensitive factor against intervention if effort for sustainability development intervened the given factor. There were 23 most influential factors in ruminant slaughterhouse, 24 attributes in chicken slaughterhouse, and 19 attributes in pig slaughterhouse (Table-5).

**Table-5:** Most influential attributes in every dimension from every slaughterhouse.

Slaughterhouse	Dimension main leverage factor				

	Social	Economy	Environment	Institutional	Technology
Ruminant	S5, S6, S7, S2, S8, S4, S9	Ec9, Ec4, Ec10, Ec2, Ec6	En5, En 8, En 9	I3, I 1	T5, T6, T3, T1
Chicken	S8, S5, S2, S9, S4, S7, S6	Ec5, Ec8, Ec4, Ec3	En 5, En 8, En 9	I 4, I 1, I 5	T5, T3, T2
Pig	S5, S6, S7, S2, S8, S9, S3	Ec5, Ec3, Ec6, Ec7, Ec9	En 5, En 8, En 9	I 1, I 2	T3, T5

### Ruminant slaughterhouse prospective test

**Figure-5:**
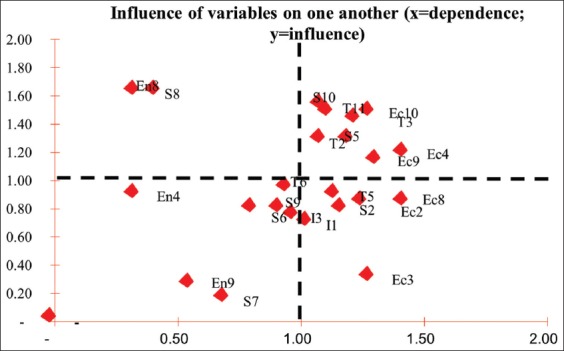
Attribute position of ruminant slaughterhouse.

Based on the prospective analysis, En8 and S8 codes were attributed with the highest influence factor and low dependability on other attributes. This means the two attributes have the highest global value out of all leverage factors (Figure-5).

Waste water usage attribute (S8) was a variable with the highest global weight so in developing sustainable ruminant slaughterhouse, waste management must be especially enforced. Waste management was the most neglected part of management in slaughterhouses, especially in a developing country. Moreover, there was a preconception that waste management required big effort and funding that it might affect whole operational cost [21]. More on the weighted global power value of ruminant slaughterhouse is provided in Table-6.

**Table-6:** Global weighted power of ruminant slaughterhouse.

Code	Leverage attributes	Global weighted power
En8	Water waste utilization (organic fertilizer/biogas)	2.96
S8	Management level of knowledge on hygiene and sanitation	2.74
T1	Waste into energy source technology	2.00
Ec10	Economic benefit for local residents	1.94
T3	Good hygiene practices implementation	1.65
S4	Slaughterhouse worker level of education	1.59
S5	Local residents’ acceptance	1.37
T2	Waste into organic fertilizer technology	1.17
En4	Level of demand	0.54
En9	Involved stakeholders (other than meat production)	0.52

### Chicken slaughterhouse prospective test

**Figure-6:**
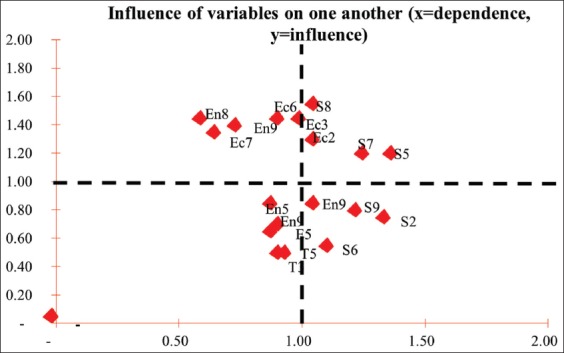
Attributes’ position in chicken slaughterhouse.

Prospective analysis test result on chicken slaughterhouse based on variable placements inside quadrants and weighted global powers showed that the main leverage or key variables in sustainable poultry slaughterhouse were attributes with code Ec5, En8, S9, and En5 (Figure-6).

In chicken slaughterhouse, promotion attribute was the main most influential attributes because the existence of chicken slaughterhouse was not well known among local people and other business stakeholders compared to other slaughterhouses. Chicken slaughterhouse has only been in operation for about 10 years (Table-7).

**Table-7:** Weighted global power of chicken slaughterhouse.

Symbol	Leverage attributes	Global weighted power
Ec5	Promotion	2.83
En8	Waste water utilization (organic fertilizer/biogas)	2.68
S7	Disease frequency in a year	2.16
En5	Waste supervision by related institution	2.09
S2	Local resident’s health problems	1.87
S9	Slaughterhouse management’s stance on hygiene and sanitation	1.54
I2	Slaughterhouse management/operation alliance	1.22
I3	Coordination with livestock supplier region	0.57

### Pig slaughterhouse prospective test

**Figure-7:**
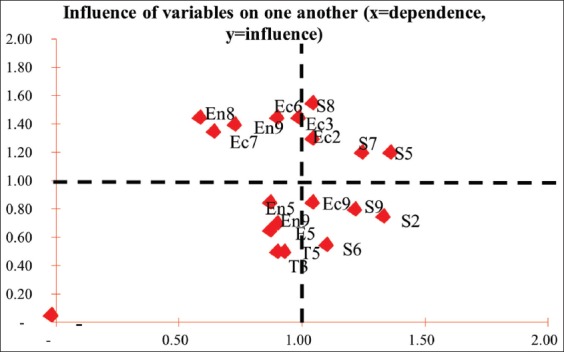
Attributes position in pig slaughterhouse.

The prospective analysis test result showed that the main leverage attribute in pig slaughterhouse was slaughterhouse product quality control (En6) and the usage of slaughterhouse waste as organic fertilizer/biogas. This was because there was a lack of veterinarians as meat health supervisor (Figure-7).

In pig slaughterhouse, slaughterhouse product quality control was the most influential factor due to the lack of supervisory units in the slaughterhouse (Table-8).

**Table-8:** Weighted global power of pig slaughterhouse.

Symbol	Leverage attributes	Weighted global power
Ec6	Slaughterhouse product quality control	2.03
En8	Waste water utilization (organic fertilizer/biogas)	1.95
Ec3	Production	1.82
S8	Management level of knowledge on hygiene and sanitation	1.74
En9	Waste threshold examination	1.66
En3	Product marketing region	1.65
En7	Livestock supply sustainability	1.50
S7	Local people disease frequency	0.98
S5	Acceptance of local people	0.82

## Discussion

### Ruminants slaughterhouse

Sustainability analysis result by MDS method in economy, institutional, and technology dimension was included as moderately sustainable with sustainability index >50. The sustainability of the three dimensions was influenced by main leverage factor, which a factor most sensitive to changes. Thus, to improve the sustainability of dimensions, treatment must be applied on leverage factor [22]. Main leverage factor in economy dimension was the involvement of other business stakeholders in slaughterhouse management other than slaughter business. The examples of these businesses are tanning, fertilizer business, and other types of business. High demand for beef in Jakarta Province could improve economy dimension only if slaughterhouse productivity is improved, as currently, the slaughterhouse could only fulfill 5.2 % of total demand [1]. The main leverage factor of institutional dimension was coordination pattern with supplier province. All animals slaughtered in the slaughterhouse came from supplier province since Jakarta is unsuitable for cow production. Coordination with livestock producing province must be maintained to ensure the continuity of livestock supply. Other than coordination, the suitability of slaughterhouse location must be in accordance with the general plan layout of DKI Jakarta Province. The main leverage factor of technology dimension was whether or not the slaughterhouse already implemented automatic, semi-automatic, or manual slaughter system. The current slaughterhouse had already implemented a semi-automatic system, which influences the quality and quantity of slaughter. The slaughterhouse also already produced meatballs, which is an example of product diversification into processed products.

Prospective test on every attribute with PPA method using questionnaire/tools different from MDS method obtained global weighted value. The global weight value is the value with the highest influence and lowest dependability level, which means that it is the main attribute that can be improved to enhance the sustainability of ruminant slaughterhouse management [23]. The highest global weight values for the slaughterhouse were utilization of liquid and solid waste into organic fertilizer where the highest potential for using slaughterhouse waste to be used as biogas is stomach waste (entire digestive tract), manure, and waste blood [21], and the level of knowledge of abattoir workers related to hygiene and sanitation has a correlation with knowledge and attitude [24]. In this ruminant slaughterhouse, 60% of slaughterhouse employee had the level of knowledge which is within good category, for sanitation, and hygiene [25].

### Poultry slaughterhouse

High demand on chicken meat in Jakarta Province could improve economy dimension only if slaughterhouse productivity is improved, as currently, the slaughterhouse could only fulfill 7.89% of total demand [1]. The sustainability of the three dimensions was influenced by main leverage factor, which a factor most sensitive to changes. Thus, to improve the sustainability of dimensions, treatment must be applied on leverage factor [22]. For poultry slaughterhouse, dimensions included as moderately sustainable was only the economy dimension with index value being >50 while other dimensions fell into less sustainable. In economy dimension, the main leverage attribute was the promotion campaign of slaughterhouse and its products and whether or not there was quality control during antemortem and postmortem. If this main attribute can be improved, the sustainability of economy dimension can increase surpassing moderate sustainability into the sustainable stage. Social, ecology, management, and technology dimension in this slaughterhouse were categorized as fairly sustainable with <50 sustainability index. To improve the sustainability status for every dimension, an improvement on main leverage factors of every dimension is required. In social dimension, improvement in employee level of knowledge and hygiene and sanitation was required since 72.7% of respondents have good knowledge and there is a clear correlation between education levels with knowledge, attitude [21]. Improvement in the local perception of slaughterhouse existence was also required since 32.6% of locals are disturbed by the existence of the slaughterhouse. In ecology dimension, the main leverage factor was the availability of waste monitoring apparatus. If waste monitoring apparatus is available and waste is utilized as organic fertilizer and biogas, the attribute will be guaranteed to improve ecology dimension’s sustainability index. In the management dimension, the main focus of poultry slaughterhouse was improving collaboration with government bodies of other region and improving the suitability of slaughterhouse location with the general layout plan of Jakarta. In the technology dimension, improving slaughter system characteristic which was still manual slaughtering into semi-automatic and improving the implementation of good hygiene practices must be the main focus of improvement.

Prospective test on poultry slaughterhouse obtained eight attributes from all dimensions (economy, ecology, social, technology, and management dimensions) with the highest global weight value. According to the prospective test, promotion attributes had the most influence with the least dependence against other attributes, after wastewater utilization (organic fertilizer/biogas) and disease frequency in a year. This might be possible for poultry slaughterhouse was still not widely known by the locals and consumers, which prompts regional government to try improving poultry slaughterhouse promotion in Jakarta City. Efforts to treat wastewater from poultry slaughterhouses can be done by adding activated carbon for the digestion of residual. Blood highly improved the digestion process. The adsorption capacity of ammonium, the protection this carrier may offer by limiting the mass transfer of toxic compounds, and its capacity to act as a conductive material may explain the successful digestion of residual blood as the sole substrate [26]. The last result of PPA in poultry slaughterhouse was disease frequency in a year which slaughterhouse waste is a potential reservoir of pathogenic, viral, prion, and parasitic bacteria that can infect humans and animals. Based on the type of liquid waste management in slaughterhouses that contain blood, protein, fat, and other solid wastes that produce organic substances can be managed through a pasteurization process using the biochemical methane potential process [27]. The liquid waste of slaughterhouses used as fertilizer for plants without going through the processing process can be a source of contamination because liquid slaughterhouses contain pathogenic microorganisms that can cause salmonellosis, leptospirosis, and tularemia [28].

### Pig slaughterhouse

The sustainability of the three dimensions was influenced by main leverage factor, a factor most sensitive to changes. To improve the sustainability of dimensions, treatment must be applied on leverage factor [22]. Leverage factors of the attributes analyzed using leverage factor analysis are obtained from the root mean square (RMS) value which is greater than the median RMS attribute that exists [29]. In pig slaughterhouse, only economy dimension was categorized as moderately sustainable with sustainability index >50, whereas other dimensions were categorized as less sustainable. In social dimension, the main leverage factor that must be improved was the acceptance of locals. Based on previous research, 96% of locals living near the slaughterhouse were disturbed by it [21]. The effort of improving slaughterhouse reception can be by better waste management to prevent disturbing the locals. The most concerning attribute in ecology dimension was the availability of waste monitoring device, such as waste water measuring device, and waste utilization effort into organic fertilizer or biogas, and currently, such efforts were yet to be found in the location. Prospective test on swine slaughterhouse found that slaughterhouse product quality control, liquid waste utilization, production improvement, and employee knowledge level on hygiene and sanitation were attributes most concerning for they have the highest global weight value. All attributes mentioned had high influence with lower dependence compared to other attributes.

## Conclusion

Pig slaughterhouse had the highest value of sustainability among the slaughterhouses with 51.73 index values, while ruminant slaughterhouse had 51.71 values and chicken slaughterhouse was lowest with 45.30. The policy scenario in ruminant slaughterhouses and pig slaughterhouses is to improve the management of slaughterhouse waste into organic fertilizer and energy sources (biogas) and increase the knowledge of abattoir managers by conducting various relevant training; chicken slaughterhouse may improve promotion through print and electronic medium or by social approaches; and pig slaughterhouse also needed to have product quality control through the addition of veterinarians.

### Authors’ Contributions

HAS supervised the present study, MM designed and coordinated the study. HAS, MM, NR, and NKP performed the experiment. HAS analyzed the data and wrote the manuscript. The final manuscript has been read and developed in consultation with all authors. All authors read and approved the final manuscript.
